# Association between Rainfall and Pediatric Emergency Department Visits for Acute Gastrointestinal Illness

**DOI:** 10.1289/ehp.0901671

**Published:** 2010-06-02

**Authors:** Patrick Drayna, Sandra L. McLellan, Pippa Simpson, Shun-Hwa Li, Marc H. Gorelick

**Affiliations:** 1 Department of Pediatrics, Medical College of Wisconsin, Milwaukee, Wisconsin, USA; 2 Great Lakes WATER Institute, Milwaukee, Wisconsin, USA; 3 Children’s Research Institute, Milwaukee, Wisconsin, USA

**Keywords:** diarrhea, environmental risk, gastroenteritis, gastrointestinal infections, public health

## Abstract

**Background:**

Microbial water contamination after periods of heavy rainfall is well described, but its link to acute gastrointestinal illness (AGI) in children is not well known.

**Objectives:**

We hypothesize an association between rainfall and pediatric emergency department (ED) visits for AGI that may represent an unrecognized, endemic burden of pediatric disease in a major U.S. metropolitan area served by municipal drinking water systems.

**Methods:**

We conducted a retrospective time series analysis of visits to the Children’s Hospital of Wisconsin ED in Wauwatosa, Wisconsin. Daily visit totals of discharge *International Classification of Diseases, 9th Revision* codes of gastroenteritis or diarrhea were collected along with daily rainfall totals during the study period from 2002 to 2007. We used an autoregressive moving average model, adjusting for confounding variables such as sewage release events and season, to look for an association between daily visits and rainfall after a lag of 1–7 days.

**Results:**

A total of 17,357 AGI visits were identified (mean daily total, 7.9; range, 0–56). Any rainfall 4 days prior was significantly associated with an 11% increase in AGI visits. Expected seasonal effects were also seen, with increased AGI visits in winter months.

**Conclusions:**

We observed a significant association between rainfall and pediatric ED visits for AGI, suggesting a waterborne component of disease transmission in this population. The observed increase in ED visits for AGI occurred in the absence of any disease outbreaks reported to public health officials in our region, suggesting that rainfall-associated illness may be underestimated. Further study is warranted to better address this association.

Waterborne disease has been associated with heavy rainfall in the United States and other parts of the world (Curriero et al. 2001; Jean et al. 2006; Miossec et al. 2000; Saidi et al. 1997; Singh et al. 2001; Weniger et al. 1983). Most of the literature has focused on reported disease outbreaks, but it is likely that such outbreaks represent only a minority of actual cases (Reynolds et al. 2008; Yoder et al. 2008). During periods of heavy rainfall, water quality may be adversely affected in several ways: contamination of surface or groundwater sources by storm water runoff from impermeable or saturated surfaces, introducing fecal contaminants including bacteria, protozoa, and viruses; cross-contamination due to infiltration and inflow between sewage and water pipes, especially in areas with aging water infrastructure; and release of sewage into local waterways because of sewage overflows or bypass (Atherholt et al. 1998; Borchardt et al. 2003; Miossec et al. 2000; Reynolds et al. 2008; Wellings et al. 1975). We previously observed an association between sewage bypass and acute gastrointestinal illness (AGI) in children (Redman et al. 2007). However, the relationship between rainfall and AGI in the absence of sewage release and in the absence of disease outbreaks reported to public health officials has not been as well described.

It is estimated that up to 19 million cases of AGI annually may be attributable to public drinking water systems in the United States (Colford et al. 2006; Messner et al. 2006; Reynolds et al. 2008). As global climate change is predicted to lead to an increase in extreme precipitation events, determining the role of weather in the incidence of waterborne disease is a public health priority (Patz et al. 2000). A better understanding of the impact that rainfall has on disease incidence is an important first step toward finding ways to mitigate risk of disease.

The aim of this study was to examine the association between rainfall and AGI in a population of children living in a major U.S. metropolitan area served by municipal drinking water systems.

## Materials and Methods

### Design

We conducted a retrospective time series analysis of rainfall and AGI visits to the Children’s Hospital of Wisconsin Emergency Department (CHW ED) in Wauwatosa, Wisconsin. The study was reviewed by the hospital’s human research review board and considered exempt.

### Setting

The study was conducted at a large freestanding pediatric hospital in southeastern Wisconsin. The CHW ED serves ~ 60,000 patients per year from a wide catchment area including urban, suburban, and rural communities. Most patients seen in the CHW ED live in communities whose water supply is surface water from Lake Michigan, provided by public utilities; the remainder use groundwater, mostly from municipal wells.

### Subjects

Over a 6-year period from 1 January 2002 to 31 December 2007, all visits to the CHW ED with discharge diagnosis of AGI were identified based on any of the following *International Classification of Diseases, 9th Revision* (ICD-9) (World Health Organization 1975) codes being recorded as the discharge diagnosis: specified gastrointestinal infections (ICD-9 codes 001-009.9), unspecified gastroenteritis (558.9), or diarrhea (787.91).

### Data collected

We collected daily visit totals of patients with discharge ICD-9 codes as above. No patient-specific data (clinical or demographic) were obtained.

Daily rainfall totals were obtained from the National Oceanic and Atmospheric Administration monitoring station at General Mitchell International Airport (National Oceanic and Atmospheric Administration–National Climatic Data Center, unpublished data). Data regarding sewage release events during the study period were obtained from the Milwaukee Metropolitan Sewerage District and Milwaukee Riverkeeper (formerly Friends of Milwaukee’s Rivers) (Milwaukee Riverkeeper, unpublished data; Milwaukee Metropolitan Sewerage District, unpublished data; Redman et al. 2007).

### Analysis

We used a type of autoregressive moving average (ARMA) model, which adjusts for confounding variables such as sewage release events and season, to look for an association between daily visits and rainfall after a defined lag. The time series of AGI visits is the response series, with a square root transformation of the number of daily visits plus 
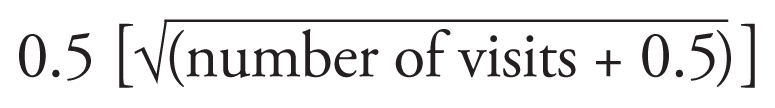
 as the outcome variable. Because the distribution of visits is slightly positively skewed, the square root transformation of visits resulted in a better fit for residuals. Further, because there were days with 0 visits, adding 0.5 helped find a model that fit well. For the main effect of rainfall, lags of 1–7 days between the date of rainfall and the date of the visit were considered. To examine the effect of extreme precipitation, defined as > 95th percentile rainfall that is > 1 inch [2.54 cm] in a 24-hr period or 1.5 inches [3.76 cm] in a 48-hr period, we performed alternate analyses incorporating these events as covariates, again examining lags from 1 to 7 days.

An event such as sewage release is considered an intervention in or an interruption of the normal evolution of the response time series, which in the absence of the intervention is usually assumed to be a pure ARMA process. An indicator variable containing discrete values flags the occurrence of an event affecting the response series. Thus for each of eight sewage releases, an indicator variable was created taking effect 3–6 days after the sewage release (Redman et al. 2007). Based on clinical considerations, the possible effects of winter months, which constitute the typical rotavirus season (December, January, February, and March), were treated as an intervention in a similar way.

The smallest canonical correlation method and extended sample autocorrelation function method are both used to tentatively identify the orders (p, q) of a stationary or nonstationary ARMA process. The notation ARMA (*p*, *q*) refers to the model with *p* autoregressive terms and *q* moving average terms. This model contains the AR (*p*) and MA (*q*) models.

We used Akaike’s information criterion (AIC) and Bayesian information criterion (BIC) to select significant lag variables and interventions. Competing models were ranked according to their AIC and BIC, with the one having the lowest AIC and BIC being considered the best. A *p*-value < 0.05 was taken as significant. Maximum likelihood methods were used in the intervention model.

We based diagnostic checks on the plots of auto-correction (ACF), inverse ACF, partial ACF, white noise probability for residuals, and residual normality plots (Q-Q plot and histogram).

Analyses were performed with SAS version 9.2 (SAS Institute Inc., Cary, NC, USA).

## Results

**Figure 1:**
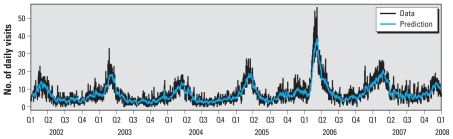
Total and predicted daily AGI visits to CHW ED from 2002 to 2008, in yearly quarters (Q).

A total of 17,357 AGI visits to the ED were identified (mean daily total, 7.9; range, 0–56) in the 2,191-day study period, with expected seasonal effect of increased visits in winter months (Figure 1). Most ED visits were from ZIP codes served by a surface water source (Table 1).

**Figure 2:**
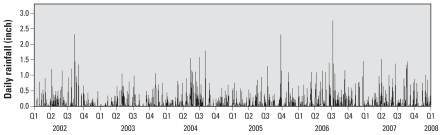
Total daily rainfall (Milwaukee County, Mitchell Airport) from 2002 to 2008, in yearly quarters (Q). Data from National Oceanic and Atmospheric Administration–National Climatic Data Center Record of Climatological Observations (unpublished data).

During the 2,191-day study period there were 776 days of rain (35.4% of days). The average daily rainfall on days with rainfall was 0.23 inches (range, 0.01–2.76) [0.58 cm (range, 0.02–7.01)], with a median of 0.1 inches (0.25 cm) (Figure 2). Extreme precipitation events [> 95th percentile defined as 24-hr rainfall > 1 inch (2.54 cm) or a 48-hr total > 1.5 inches (3.81 cm)] occurred 37 times. There were eight sewer overflows > 1 million gallons (3,785,412 L) reported during this period.

### Regression analysis

Among all candidate models, the ARMA(1,1) model with interventions sewage event 3 and winter effect has the best fit because of smallest values of both AIC and BIC. The visits predicted by the model are shown in Figure 1. We examined rainfall lags from 1 to 7 days, eight combined sewer overflow events, and a seasonal effect. The results of the full and reduced ARMA regression models are shown in Table 2. Expected seasonal effects were seen, with increased AGI visits in winter months. One of the eight sewer overflows, occurring in December 2003 with an estimated volume of 39 million gallons (147,631,100 L), was also significantly associated with increased visits. This was the only sewage overflow to occur during the winter. To determine whether the effect of rainfall might depend on the presence of sewer overflow events, we examined models with these events removed, and the estimated effect of rainfall was unchanged. In addition, when extreme precipitation events were modeled, there was no significant association with ED visits at any lag between 1 and 7 days.

Of the lags tested, only rainfall 4 days prior was significantly associated with number of visits. The test of model fit suggests that the relationship between outcome and ED visits was linear. However, because the outcome was a square-root transformation of visits in the model, the clinical relevance of the coefficients is difficult to interpret intuitively, and the increase in visits per unit increase of rainfall cannot be calculated directly. To provide some estimate of clinical importance, we therefore dichotomized each day as either rain or no rain and calculated average visits (and percent change in visits) predicted from the model after a lag of 4 days (Table 3). The estimated mean number of visits 4 days after any rainfall is 8.1 [95% confidence interval (CI), 7.2–8.5], which is 11% higher than the estimated mean of 7.3 visits (95% CI, 7.1–7.6) 4 days after days without rainfall. As anticipated, this apparent effect is smaller than the effect of winter month (120% relative increase). Results were still unchanged when visits were restricted to patients residing in areas served by surface water only (85% of total visits).

## Discussion

We observed a statistically significant association between rainfall and pediatric ED visits for AGI, with an estimated 11% increase in visits 4 days after rainfall. The etiology of AGI in children is due largely to viral agents (mainly rotavirus, norovirus, and other enteric viruses such as enterovirus, calicivirus, and adenovirus) which have typical incubation times from 1 to 7 days (American Academy of Pediatrics 2006; Elliott 2007). Bacterial causes of AGI (such as *Campylobacter sp.*, *Salmonella sp.*, and *Escherichia coli sp.*), although reportable and much less common, also have similar incubation times on the order of one to several days. Although protozoan causes such as *Giardia* and *Cryptosporidium* can have longer incubation times than 7 days, these organisms were specifically screened per water treatment protocol. Thus, we felt that given the suspected timing of exposure from rainfall and incubation of the likely infectious agents, a lag window of 1–7 days from rainfall would be appropriate for investigation.

Further, one previous study showed the risk of treatment failure (defined as the presence of fecal coliforms) of drinking water supplies was linearly associated with rainfall on the prior day (Richardson et al. 2009); another found a lag of 1–2 days between increased water turbidity and self-reported gastrointestinal illness (Egorov et al. 2003). Increased water turbidity levels in the drinking water of Philadelphia, Pennsylvania, have been associated with increased pediatric AGI visits to the ED 4 days later as well (Schwartz et al. 1997). An association between raw water turbidity and ED visits for gastrointestinal illness in Atlanta, Georgia, has also been shown (Tinker et al. 2008). Data from an ongoing study of the epidemiology of AGI in our ED show that patients have symptoms for an average of 2–3 days prior to the visit (Gorelick M, unpublished data). Thus, the observed 4-day time lag in our study is consistent with the expected timing of exposure.

Our results add to the current knowledge that AGI outbreaks reported to health officials are often associated with rainfall by demonstrating that unreported, endemic AGI is associated with rainfall as well. A review of 548 disease outbreaks reported to the U.S. Environmental Protection Agency between 1948 and 1994 found a significant association between rainfall and illness, with 68% of the events preceded by precipitation events above the 80th percentile (Curriero et al. 2001). Investigators in Canada found that in the last quarter of the 20th century, rainfall events above the 93rd percentile increased the risk of a waterborne disease outbreak by a factor of 2.3 (Thomas et al. 2006). Disease outbreaks after heavy rainfall have been attributed to a variety of pathogens, including *Cryptosporidium* (Atherholt et al. 1998; MacKenzie et al. 1994), *Giardia* (Atherholt et al. 1998; Weniger et al. 1983), and enterovirus (Jean et al. 2006). What is novel about our findings is that the observed increase in ED visits for AGI occurred in the absence of any outbreaks reported to public health authorities in our region, suggesting that rainfall-associated illness may be underestimated. We were unable to demonstrate any additional effect of extreme rainfall. Most likely this is attributable to the relatively small number of extreme precipitation events during the study period.

The association between rainfall and pediatric ED visits for AGI in our population is plausible, given the presence of pathogens that are detectable in surface and groundwater at baseline levels that increase after rainfall (Signor et al. 2005). Corsi et al. demonstrated that adenovirus, enterovirus, norovirus, hepatitis A virus, and rotavirus have all been detected in over half of water samples from local waterways in the Milwaukee River watershed and significantly increase in concentration after storm runoff events (Corsi S, Hughes P, Borchardt M, Spencer S, Baldwin A, unpublished data). Several surveillance studies of groundwater systems across the United States have demonstrated infiltration of viral pathogens (Abbaszadegan et al. 2003; Borchardt et al. 2003, 2004; Fout et al. 2003), which suggests that these drinking water sources are also vulnerable to the effects of AGI pathogen introduction from rainfall and surface water contributions.

Drinking water may become contaminated by multiple routes, including ineffective treatment of source water affected by sewage discharges or through breeches in the distribution system that allow contaminated water to enter. Many cities around the Great Lakes, including Milwaukee, are served by combined sewer systems that can become inundated with rainwater and release untreated sewage. The vast majority of patients in our region live in households served by municipal water utilities that follow state and federal treatment recommendations and whose water meets all current standards (Milwaukee Water Works 2008; U.S. EPA 2002, 2006). Disease transmission due to upstream contamination of the water sources should therefore be prevented or mitigated by treatment. However, municipal water systems may be overwhelmed during heavy rainfall events (Curriero et al. 2001). Moreover, although procedures for wastewater treatment are effective in reducing concentrations of enteric pathogens and quality is tested by monitoring for fecal coliform indicator bacteria, *Giardia*, *Cryptosporidium*, and chemical contaminants, the effectiveness of treatment in removing viral pathogens is unclear (Reynolds et al. 2008), and viral testing is not routinely done. Failure of such indicator bacteria standards to reflect the occurrence of enteric viruses has been described previously (Borchardt et al. 2004; Gerba et al. 1979). It is also possible that contamination of drinking water may occur after leaving water treatment facilities because of infiltration and inflow in areas of aging infrastructure where clean water and sewage pipes run in close proximity to each other and are made of porous material (Hunter et al. 2001, 2005; LeChevallier et al. 2003; Tinker et al. 2009). The observed association of rainfall and illness was independent of combined sewer overflows that occurred during excessive rainfall. Indeed, only one of the sewer overflow events during this study period was independently associated with increased AGI visits, suggesting that contamination of source water by sewage release is not the most likely mechanism for exposure and that more complex pathways are involved. Interestingly, this was the only sewage overflow event that took place during winter. Although the significance of this is unclear, it is possible that contaminated runoff is greater when the ground is frozen or that the rapid freeze/thaw cycle that occurred would have compromised distribution system integrity in a more severe manner than usual. This is speculative but plausible, and consistent with a suggested mechanism of post-treatment facility contamination via an aging distribution infrastructure.

The importance of this association between rainfall and pediatric AGI is underscored by the fact that global climate change is expected to increase the intensity and frequency of extreme precipitation events (Patz et al. 2000, 2008). With further study, the health impact of such changes could be mitigated by changes in monitoring or interventions such as changes in water treatment and delivery systems practices or issuing boil alerts around periods of heavy rain.

Our study is limited by the lack of individual data regarding disease etiology, clinical course, drinking water source and habits, and recreational water exposures. We also used only daily precipitation totals from a single weather station and did not have data on precipitation intensity, potentially masking localized effects. In addition, because of the relatively small number of visits from ZIP codes served by groundwater, we did not attempt to analyze the data by this water source, which may be an important factor in linking AGI risk to specific types of municipal water systems.

Our data include ED visits only, which could skew the data based on severity of illness. However, if the latter is true, our results would then likely reflect an underestimation of the true incidence of disease. Random-digit dialing or cohort follow-up studies may be more effective methods in assessing community-wide incidence of disease. The use of ED data gives consistency in referencing one data set for the community, and as mentioned above, ours is the only pediatric ED in the region. Furthermore, ED data have been used as a uniform sentinel for community-wide events of gastrointestinal illness in similar contexts before (Schwartz et al. 1997; Tinker et al. 2009).

## Conclusion

In summary, we demonstrate a significant association between rainfall and pediatric ED visits for AGI, suggesting a waterborne component of disease transmission in this population. A better understanding of the impact that rainfall has on this burden of disease is an important first step in finding ways to mitigate risk of disease, especially in an era of climate change. Further study is warranted to better address this association and potential mechanisms for introduction of pathogens into environmental and drinking waters.

## Figures and Tables

**Table 1:** CHW ED visits for AGI (by water source) 2002–2008.

Water source	No. of AGI visits	Mean no. of daily visits	Range	Percent of total visits
Total	17,357	7.9	0–56	100
Surface water	14,767	6.7	0–50	85.1
Groundwater	2,067	0.94	0–8	11.9
Mixed or unknown	523	0.24	0–6	3.0

**Table 2:** Regression estimates from ARMA models.

Variable	Lag	Coefficient	SE	*p*-Value
Full model				
Square root of total visits for date	Mean	2.650	0.209	< 0.0001
	MA1	0.805	0.014	< 0.0001
	AR1	0.989	0.003	< 0.0001
Total rainfall for date	0 days	−0.070	0.053	0.19
	1 day	0.022	0.053	0.67
	2 days	−0.001	0.053	0.99
	3 days	−0.059	0.054	0.27
	4 days	0.138	0.054	0.01
	5 days	0.019	0.054	0.73
	6 days	0.043	0.054	0.42
	7 days	−0.079	0.055	0.14
Sewer overflow				
7 April 2002		−0.107	0.314	0.73
12 August 2002		0.061	0.320	0.85
10 December 2003		0.940	0.313	0.003
13–25 May 2004		−0.229	0.313	0.46
3–4 April 2007		0.087	0.242	0.71
18–20 August 2007		0.162	0.312	0.60
10–11 April 2008		−0.131	0.3294	0.66
7–16 June 2008		0.084	0.245	0.73
Winter (December–March)		0.284	0.095	0.003

Reduced model				
Square root of total visits for date	Mean	2.653	0.208	< 0.0001
	MA1	0.805	0.014	< 0.0001
	AR1	0.989	0.003	< 0.0001
Total rainfall for date	0 days	−0.068	0.052	0.20
	1 day	0.023	0.052	0.66
	2 days	0.002	0.052	0.97
	3 days	−0.059	0.052	0.26
	4 days	0.140	0.052	0.008
Sewer overflow (10 Dec 2003)		0.926	0.311	0.003
Winter (December–March)		0.284	0.095	0.003

Abbreviations: AR, auto-regressive; MA, moving average.

**Table 3:** Model-predicted means for grouped variables of interest.

Variable	Class	*n* Obs	Mean	95% CI
Rainfall 4 days later	No rain	1,417	7.3	7.1–7.6
	Rain	774	8.1	7.2–8.5
Sewage release on 10 December 2003	No	2,187	7.6	7.4–7.8
	Yes	4	9.5	9.0–10.0
Winter (December–March)	No	1,464	5.5	5.4–5.7
	Yes	727	11.8	11.4–12.3

*n* Obs, no. of observations.
